# A Hypercalcemic Deception: Uncovering an Unusual Case of Familial Hypocalciuric Hypercalcemia

**DOI:** 10.7759/cureus.95514

**Published:** 2025-10-27

**Authors:** Shruti M Gandhi, Eric S Nylen

**Affiliations:** 1 Endocrinology, Veterans Affairs Medical Center, Washington DC, USA; 2 Endocrinology and Diabetes, Veterans Affairs Medical Center, Washington DC, USA; 3 Endocrinology, George Washington University School of Medicine and Health Sciences, Washington DC, USA

**Keywords:** familial hypocalciuric hypercalcemia, hypercalcemia, hyperparathyroidism, missense mutation, thyroid cancer

## Abstract

Although a rare cause of hypercalcemia, familial hypocalciuric hypercalcemia (FHH) is an inherited condition most often due to a missense mutation in the calcium-sensing receptor (CASR) gene, giving rise to increased calcium levels with elevated parathyroid hormone (PTH) levels and hypocalciuria. Many clinical features of FHH show a high degree of overlap with the much more common disorder of primary hyperparathyroidism (PHPT), making the correct diagnosis a challenge since surgery should be avoided in FHH. In the current case, although PHPT was initially suspected based on urine biochemistry and imaging, FHH-1 and metastatic micropapillary thyroid cancer were diagnosed following thyroid surgery. Moreover, an underlying novel missense CASR mutation shared with the patient’s biological father was uncovered. This case highlights the challenge of making the correct hypercalcemic diagnosis without genetic tools. The concurrence with thyroid cancer has been reported once before, albeit not with this novel CASR mutation.

## Introduction

Among causes of hypercalcemia, familial hypocalciuric hypercalcemia (FHH) is a rare condition that is inherited in an autosomal-dominant fashion, giving rise to chronic hypercalcemia, hypocalciuria, and normal to mildly elevated parathyroid hormone (PTH) levels [1]. In the majority of cases, the underlying cause is a loss-of-function missense mutation in the calcium-sensing receptor (CASR) gene, now termed FHH type 1, impairing its receptor function [2]. Less common causes of FHH include a guanine nucleotide-binding protein subunit alpha 11 (GNA11) gene abnormality termed FHH type 2, which impacts PTH release, as well as an endocytosis abnormality that occurs with an adaptor-related protein complex 2 sigma subunit 1 (AP2S1) gene mutation termed FHH type 3 [3,4].

By far the most common hypercalcemic condition with elevated PTH is primary hyperparathyroidism (PHPT). Both PHPT and FHH have overlapping benign and often asymptomatic clinical features. Moreover, the proposed diagnostic biochemistry focused on urinary calcium handling, i.e., the calcium creatinine clearance ratio, is commonly misleading [5]. Importantly, these two conditions need to be distinguished since only hyperparathyroidism (HPTH) may be remedied by surgery. Indeed, about 10-23% of parathyroidectomies deemed to be failures are due to mistaken diagnosis of PHPT instead of FHH [6,7]. Separating PHPT from FHH, as in the current case, can thus be a significant challenge, and those patients who are younger and/or those with a family history of parathyroid abnormalities require more in-depth evaluation, including genetic testing, to avoid a mistaken diagnosis.

## Case presentation

A 37-year-old White veteran male was referred for hypercalcemia of 10.9 mg/dL (Table 1) and elevated PTH of 115 pg/mL. The patient was asymptomatic and denied kidney stones, and had normal kidney function and normal bone density by dual-energy X-ray absorptiometry. Magnesium and bone markers (alkaline phosphatase) were normal. Family history was positive for hypercalcemia. The biological father was diagnosed with asymptomatic hypercalcemia with elevated PTH at age 48, and despite partial parathyroidectomy (no adenoma present), hypercalcemia persisted. Moreover, the paternal aunt also had a mild hypercalcemia status post partial parathyroidectomy.

**Table 1:** Laboratory values. PTH: parathyroid hormone; Cl/Phos ratio: chloride-to-phosphate ratio; Ca: calcium; FECa: fractional excretion of calcium.

Labs (reference range)	Calcium (8.9-10.5 mg/dL)	Vitamin D (30-100 ng/mL)	PTH (15-65 pg/mL)	Cl/Phos ratio (<33)	24-hour urine Ca (55-300 mg/24 hr)	FECa
Initial	10.9 mg/dL	27.2 ng/mL	114.6 pg/mL	34	392 mg/24 hr	0.014
Postoperative	9 mg/dL	32 ng/mL	90 pg/mL	32.19	201 mg/24 hr	0.007

**Figure 1:**
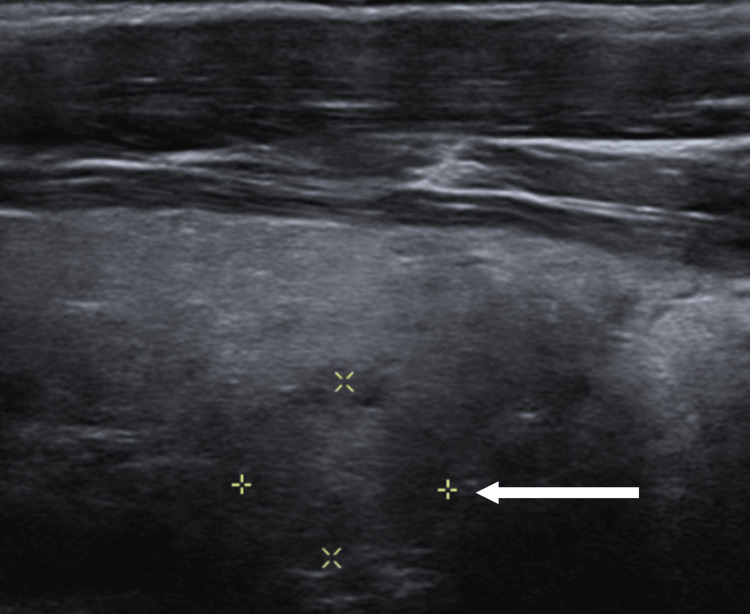
Neck imaging. Neck ultrasound showing a 1.2 x 1 x 1 cm left nodule (arrow), interpreted as a possible parathyroid adenoma.

**Figure 2:**
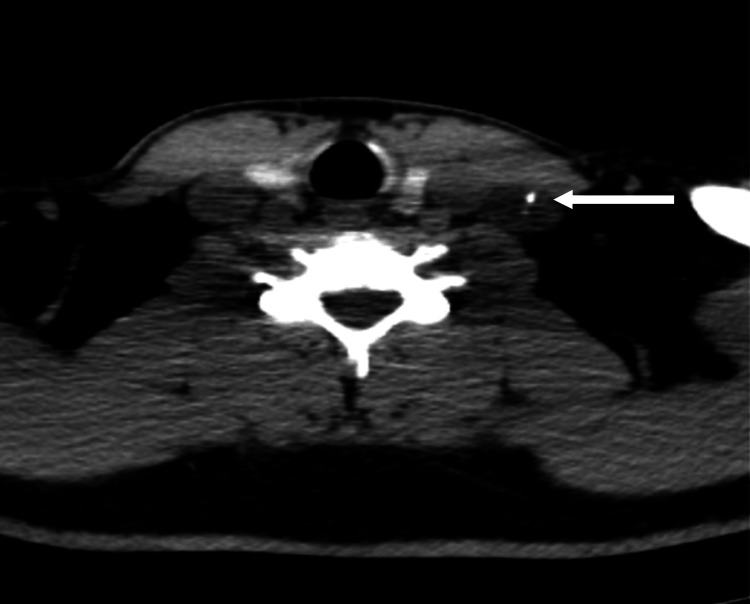
Neck imaging. Sestamibi scintigraphy showing a possible parathyroid adenoma measuring 1.5 x 0.7 x 0.4 cm in the left thyroid.

The initial laboratory evaluation is shown in Table 1, with a normal vitamin 25(OH)D, an elevated chloride-to-phosphate ratio of 34, and elevated 24-hour urinary calcium, while urinary fractional excretion of calcium was 0.014. Neck ultrasound imaging showed a 1.2 x 1 x 1 cm left nodule, considered a possible parathyroid adenoma, which was further supported by positive 99mTc-Sestamibi scintigraphy (Figures 1, 2).

**Figure 3:**
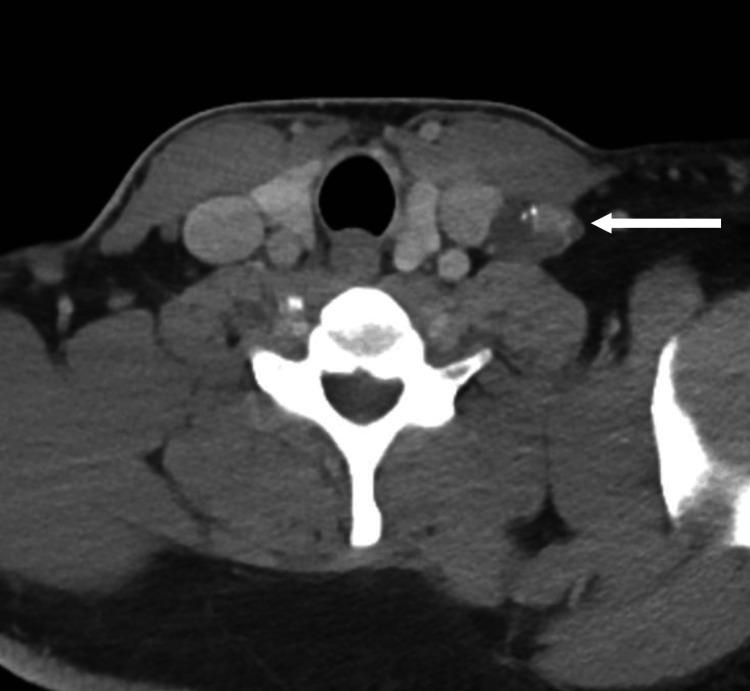
Neck imaging. CT scan showing left middle pole thyroid nodule (arrow) as well as a possible lymph node with calcifications.

Neck CT imaging demonstrated a well-defined polypoid lesion with calcification at the left level IV neck, raising concern for carcinoma (Figure 3). During fine needle aspiration of the left neck mass, a small sub-centimeter thyroid nodule on the isthmus was also demonstrated and aspirated. Aspiration of the level IV mass was found to be metastatic papillary thyroid cancer, described as cohesive sheets and clusters of atypical follicular cells with enlarged, elongated, and irregular nuclei and numerous intranuclear inclusions. The isthmus nodule was found to be benign. The patient underwent total thyroidectomy and subtotal parathyroidectomy with lateral neck dissection. On final pathology, the thyroid exhibited multifocal (six foci) micropapillary thyroid cancer with focal tall cell and clear cell features (T3N1b) and BRAF V600 positivity. Extrathyroidal extension included 6/39 positive unilateral lymph nodes (III, IV, and VI, the largest being 3.3 cm). In addition, parathyroid benign hyperplasia (i.e., multi-glandular parathyroid disease) was also documented.

Calcium levels normalized postoperatively, as shown in Table 1; however, PTH rose again. Urinary work-up showed a fractional excretion of calcium of 0.007, suggesting FHH. Genetic testing (hyperparathyroidism panel, Invitae Corp., San Francisco, CA) revealed a heterozygous CASR gene missense mutation: c.1382T>C (p.Leu461Pro), a variant of uncertain significance [8]. Upon further inquiries, the biological father had been diagnosed with the same CASR mutation variant and had a low fractional calcium excretion of 0.004. Further genetic testing for MEN1, RET, AP2S1, CDC73, CDKN1B, and GNA11 in the index patient and father was negative.

## Discussion

Hypercalcemia is a common clinical occurrence dominated by HPTH and malignancy, although it is not associated with thyroid cancer outside of very rare cases of anaplastic thyroid cancer [9]. The current hypercalcemic case highlights the difficulty in distinguishing PHPT from FHH, which was only apparent following surgery, which uncovered a novel CASR mutation and unsuspected metastatic micropapillary thyroid cancer. The initial biochemical and imaging findings suggested PHPT with the initial fractional excretion of calcium ratio in the “gray zone” above 0.01. Usually, a fractional excretion of calcium ratio > 0.01 is suggestive of HPTH, but as many as 20% of PHPT cases have ratios <0.01 [5,10]. In contrast, 20-35% of those with FHH can have a ratio >0.01 [11]. The proposed fractional excretion of calcium ratio cut-off at or below 0.020 suggests a diagnostic sensitivity of 98% for having FHH when performed with concurrent genetic testing [6,12]. In a recent surgical series tracking FHH by genetic analysis, 38% had a urinary calcium of >100 mg, and 23% had a calcium ratio >0.01 with a suboptimal resultant receiver operating ratio and unnecessary surgery [13]. It is also important to monitor other variables that can falsely influence the urinary ratio, including low calcium intake and vitamin D status (falsely low in PHPT) [14]. Likewise, the misleading 99mTc-Sestamibi scan, which was a false positive in the current case, is a finding reported in the setting of thyroid adenomas, lymph nodes, diffuse hyperplasia, brown adipose tissue activity, and metastatic thyroid cancer due to prolonged mitochondrial retention [15].

The most common CASR abnormality involves single amino acid missense mutations in the long arm of chromosome 3 at 3q21.1, which reduces the receptor function of this 1078 amino acid protein. The genetic testing in our case confirmed FHH-1, one of 377 reported missense mutations so far reported for FHH-1 [16]. This missense mutation occurring at a conserved site of the extracellular domain of exon 5 was also documented in his father and was reported in two prior cases [3]. Interestingly, protein-modifying mutations involving a proline residue replacing leucine, as in the current case, have been reported in several pathological circumstances [17,18]. The CASR is expressed in a variety of tissues (e.g., parathyroid, kidney, bone, thyroidal C cells, and enterocytes), and disorders occasionally associated with FHH include concurrent PHPT, chondrocalcinosis, pancreatitis, nephrocalcinosis, and shortened QT on ECG. FHH-1 is most often asymptomatic, but concurrent chondrocalcinosis, osteoporosis, and nephrolithiasis have been reported [19]. However, concurrent thyroid cancer and FHH has only been reported once before [20], so the significance of this association is unknown.

## Conclusions

FHH is a rare, asymptomatic, non-surgical, hypercalcemic condition that is a challenge to distinguish from the much more predominant, and surgically remediable, PHPT. In the current case, it was perhaps fortuitous that a misleading diagnosis of PHPT was initially made since it led to surgery and the discovery of thyroid cancer. The implication is that of the increased importance of genetic testing in FHH diagnosis. The presence of a novel CASR mutation with thyroid cancer in this case is of unknown significance and can only be resolved in long-term follow-up and future studies.
